# Compliance with telephone triage advice among adults aged 45 years and older: an Australian data linkage study

**DOI:** 10.1186/s12913-017-2458-y

**Published:** 2017-08-01

**Authors:** Duong Thuy Tran, Amy Gibson, Deborah Randall, Alys Havard, Mary Byrne, Maureen Robinson, Anthony Lawler, Louisa R. Jorm

**Affiliations:** 1 Centre for Big Data Research in Health–Faculty of Medicine, UNSW Sydney (The University of New South Wales), Sydney, NSW 2052 Australia; 2 Healthdirect Australia, 133 Castlereagh Street, Sydney, NSW 2000 Australia; 3 School of Medicine, University of Tasmania and Healthdirect Australia, Department of Health and Human Services, Level 2, 22 Elizabeth Street, Hobart, TAS 7000 Australia

**Keywords:** Australia, Compliance, Healthdirect helpline, Older patients, Telephone triage

## Abstract

**Background:**

Middle-aged and older patients are prominent users of telephone triage services for timely access to health information and appropriate referrals. Non-compliance with advice to seek appropriate care could potentially lead to poorer health outcomes among those patients. It is imperative to assess the extent to which middle-aged and older patients follow triage advice and how this varies according to their socio-demographic, lifestyle and health characteristics as well as features of the call.

**Methods:**

Records of calls to the Australian healthdirect helpline (July 2008–December 2011) were linked to baseline questionnaire data from the 45 and Up Study (participants age ≥ 45 years), records of emergency department (ED) presentations, hospital admissions, and medical consultation claims. Outcomes of the call included compliance with the advice “Attend ED immediately”; “See a doctor (immediately, within 4 hours, or within 24 hours)”; “Self-care”; and self-referral to ED or hospital within 24 h when given a self-care or low-urgency care advice. Multivariable logistic regression was used to investigate associations between call outcomes and patient and call characteristics.

**Results:**

This study included 8406 adults (age ≥ 45 years) who were subjects of 11,088 calls to the healthdirect helpline. Rates of compliance with the advices “Attend ED immediately”, “See a doctor” and “Self-care” were 68.6%, 64.6% and 77.5% respectively, while self-referral to ED within 24 h followed 7.0% of calls. Compliance with the advice “Attend ED immediately” was higher among patients who had three or more positive lifestyle behaviours, called after-hours, or stated that their original intention was to attend ED, while it was lower among those who lived in rural and remote areas or reported high or very high levels of psychological distress. Compliance with the advice “See a doctor” was higher in patients who were aged ≥65 years, worked full-time, or lived in socio-economically advantaged areas, when another person made the call on the patient’s behalf, and when the original intention was to seek care from an ED or a doctor. It was lower among patients in rural and remote areas and those taking five medications or more. Patients aged ≥65 years were less likely to comply with the advice “Self-care”. The rates of self-referral to ED within 24 h were greater in patients from disadvantaged areas, among calls made after-hours or by another person, and when the original intention was to attend ED. Patients who were given a self-care or low-urgency care advice, whose calls concerned bleeding, cardiac, gastrointestinal, head and facial injury symptoms, were more likely to self-refer to ED.

**Conclusions:**

Compliance with telephone triage advice among middle-age and older patients varied substantially according to both patient- and call-related factors. Knowledge about the patients who are less likely to comply with telephone triage advice, and about characteristics of calls that may influence compliance, will assist in refining patient triage protocols and referral pathways, training staff and tailoring service design and delivery to achieve optimal patient compliance.

**Electronic supplementary material:**

The online version of this article (doi:10.1186/s12913-017-2458-y) contains supplementary material, which is available to authorized users.

## Background

Australia’s population, like that of most other developed countries, is ageing [1, 2]. The proportion of people aged ≥65 years in Australia increased from 11.8% to 14.7% between 1994 and 2014 and is projected to grow faster over the next decades [2]. The ageing population has significant implications for the health system [1], including accelerating demand for emergency department (ED) services [3–5]. Between 45% and 60% of low acuity attendances at ED among Australian patients aged 65–80 years could potentially be managed in primary care settings [6].

Telephone triage and advice services feature among strategies to manage demand for health care services and to facilitate equity of access [7, 8]. These services have proliferated in Australia, the United States, Canada, New Zealand, the United Kingdom and other European countries, and are typically delivered by experienced nurses who refer patients to the most appropriate level of care. Recent systematic reviews [9, 10] found that patient compliance with triage advice varied, with higher compliance in patients receiving advice to self-care (pooled rate 78.9%, median 77%) or to attend ED (pooled 63%, median 75%), and lower compliance among those advised to seek primary care (pooled 44%, median 66%). Some studies have also examined patient non-compliance, particularly in patients who self-refered to the ED despite receiving advice not to do so, with rates ranging between 1% to 9% [9–11].

Compliance with telephone triage advice is generally measured either by self-report or through linkage to service utilisation or claims data [9, 10, 12]. Although follow-up surveys or phone interviews are able to identify the reasons why patients did not follow the advice provided, these study designs are subject to bias in self-report of compliance [13, 14] and relatively small sample size [12, 13, 15, 16]. Record linkage studies potentially offer objective measurement of compliance over a longer time span than surveys. Most linkage studies have focused on paediatric patients [17–22], callers to triage services embedded in health insurance or health management organisations [23–25], and those who called general practices for a same-day consultations or appointments [26, 27]. Among three studies [11, 28, 29] that have reported compliance among patients calling publicly funded triage services, only the Canadian evaluation of the Health Link Alberta service [29] examined compliance with emergency, office, and self-care advice through comprehensive data linkage at population level. The other two studies, which evaluated healthdirect helpline patients in Western Australia [11] and NHS Direct patients in Southwest London [28], examined compliance only with emergency care advice, and both were restricted to small geographic areas [11, 28]. To date, no study has been conducted with a focus on compliance among middle-aged and older patients, who are more likely to require complex care due to morbidity, functional limitation, greater risks of symptom deterioration and poorer access to health and other services [1, 2].

Patients’ compliance is likely to be influenced by multiple factors, including the patient’s self-assessment about the level of care needed, their social circumstances, the quality of communication between patients and triage staff, and the availability and accessibility of the services to which they are referred [9–11, 26, 30]. Despite the growth of telephone triage as a means of unscheduled health care delivery, research evidence on factors influencing patient action after triage is patchy, due in part perhaps to the limited patient information that can be recorded at the time of calls. It has been reported that compliance increases with patient satisfaction with the advice [9, 12] and when the triage advice matches the patient’s expectation for care [10]. In the Canadian data linkage study [29], patients aged four years or older, living in higher income areas, or having better health status were more likely to comply with advice to attend emergency or primary care. Patient compliance was found to vary according to triage protocols [29], for example, subjects with respiratory symptoms were less likely to follow the advice to go to ED or to self-care, compared to those with cardiac problems. In contrast, patient non-compliance could be explained by changes in their symptoms, misunderstanding or choosing to ignore the triage advice [9, 10, 19]. Given that most of the previous studies have included children only [17–22] or patients of all ages [9, 10, 13, 15, 26–29], further investigation of factors associated with adherence among people of middle age and older has the potential to inform the planning and delivery of services.

In Australia, the healthdirect helpline was established by the National Health Call Centre Network in 2006 to provide access to health information and advice 24 h a day, 7 days a week. The healthdirect helpline is staffed by registered nurses and receives approximately 1,000,000 calls annually. In July 2011, the healthdirect helpline was extended to include an after hours GP helpline to receive calls transferred by the triage nurses for further assessment [31]. This study linked records of the healthdirect helpline and after hours GP helpline calls to comprehensive questionnaire data from a large-scale cohort study, and administrative health services data collections to assess the extent to which middle-aged and older patients comply with telephone triage advice, and how this varies according to patient socio-demographics, lifestyle behaviours, health status, and characteristics of the calls.

## Methods

### Study design, data sources and linkage

This was an observational follow-up study, using record linkage. The study subjects were participants in the 45 and Up Study [32] who had been the subject of a call to the healthdirect helpline between July 2008 and December 2012.

#### The 45 and up study

The 45 and Up Study is a cohort study of people aged 45 years and older living in New South Wales (NSW) Australia and is managed by the Sax Institute [33]. Prospective participants in the 45 and Up Study were randomly sampled from the enrolment database of Medicare Australia (now the Department of Human Services)– the universal health insurance program for all Australian residents and eligible visitors – with oversampling of people aged 80 years and older and residents of rural and remote areas. A total of 267,153 participants joined the Study between January 2006 and December 2009 by completing a baseline questionnaire (response rate 18%) and giving signed consent for follow-up and linkage of their information to routine health databases [33]. The 45 and Up Study baseline questionnaire was linked to the following data sources:

(i) healthdirect helpline calls (July 2008–December 2012), including calls transferred to the after hours GP helpline
(ii) Medicare Benefits Schedule (MBS) claims for medical consultations (January 2006 – December 2011)
(iii) ED data collections in NSW and the Australian Capital Territory (ACT) (January 2006 – June 2013), and
(iv) Hospital admission data collections in NSW and ACT (January 2006 – June 2013).

NSW is Australia’s most populous State with more than 7.5 million residents as of June 2014 [2]. The ACT (population of 385,996) is geographically surrounded by NSW. It is not uncommon for NSW citizens living near the border to utilise ACT hospital services. Although this study included only participants who were NSW residents, records of ED and hospital data for both NSW and the ACT were linked to capture the use of those services in the ACT.

#### Healthdirect helpline call

The healthdirect helpline triages patients using a computerised clinical decision support system (CareEnhance Call Centre Software) which incorporates approximately 400 standardised guidelines. In addition to recording the patient’s health concerns, the triage nurse also obtains demographic details including age and sex of both the patient and the caller, the relationship of the caller to the patient, and postal code. At the completion of the triage process, the nurse provides the patient with one of the following dispositions:

(i) direct transfer to ambulance services
(ii) attend ED immediately
(iii) see a doctor either immediately or within a specific time frame (4 h, 24 h, 72 h or 2 weeks)
(iv) self-care advice or health information only, and
(v) see a dentist or other health provider within a specific time frame.

The nurse can also refer the patient to a Poisons Information Centre, the Medicines Line, nursing posts in remote areas, acute mental health services, or transfer the caller to the after hours GP helpline for further assessment. Dispositions given by the after hours GPs include those that are similar to the nurse’s dispositions (i) to (iv), and further include the advice “self-care until seeing a GP or a health provider in-hours”. During the triage, the callers are also asked about their original intention i.e. what they intended to do prior to contacting the helpline.

#### MBS claims

The Australian Department of Human Services processes MBS claims to subsidise fees for patients who attend eligible registered health professionals [34]. For this study, medical attendances were identified using MBS item numbers as per scheduled Groups A1-A8, A11-A26, A28, A30, M2 and M12 [34]. As MBS claims data provide only date (not time) of services, an algorithm (Additional file 1: Table S1) was developed for this study to classify consultations as occurring before or after the call. The algorithm took into account factors including presence of a MBS claim, ED visit or hospital admission (on the same day of the call or subsequent day), category of MBS items (specific to the after-hours period or generic items non-indicative of consultation time), time of the call, time of arrival and discharge from ED or hospital, and constant parameters (10-min duration of triage call, 20-min duration of medical consultation, and 20-min between call and arrival at ED or hospital).

#### ED presentation and hospital admission collections

The NSW ED data include presentations to EDs in public hospitals. Of a total 150 EDs in NSW, all large EDs participate in the data collection, with the number of participating EDs increasing over time (89 in 2008 to 130 in 2012) [35]. In 2013, the ED data collection covered about 96% of all ED attendances in NSW [36]. The NSW hospital admission data include all records of hospital separations (discharges, transfers and deaths) in all public and private hospitals. In the ACT, the ED and hospital records were extracted from one of the two major hospitals (records from the other hospital were not available).

#### Data linkage

The Sax Institute deterministically linked MBS claims to the 45 and Up Study baseline questionnaire using an encrypted unique random number. All other datasets were linked by the NSW Centre for Health Record Linkage using a probabilistic matching method [37] and the privacy preserving approach [38]. The validity of the probabilistic record linkage is extremely high, with false positive links ≤0.4% [37]. De-identified datasets were provided to the researchers.

### Study outcomes

This study examined four outcomes of healthdirect calls (further defined in Table 1), including:

(i) compliance with the disposition “Attend ED immediately”,
(ii) compliance with the dispositions “See a doctor immediately, within 4 hours or 24 hours”,
(iii) compliance with the disposition “Self-care”; and
(iv) self-referral to ED or hospital within 24 h of the call for which the patients were given the “Self-care” or low-urgency dispositions.

**Table 1:** Definition of study outcomes

Study outcomes	Definition
Compliance: Attend ED immediately	Presence of an ED or hospital record within 24 h of call
Compliance: See a doctor immediately, in 4 h or 24 h	Presence of an ED or hospital record within 24 h of call or a MBS claim within 2 days
Compliance: Self-care	No ED or hospital record within 48 h of call and no MBS claim within 2 days
Self-referral to ED or hospital in 24 h	Presence of an ED or hospital record within 24 h of call among patients who were given the disposition “self-care” or low-urgency dispositions (including “see doctor in 72 h or 2 weeks”, “see a dentist in 72 h, in 2 weeks or when available”, and “see a health provider in 72 h, in 2 weeks or when available”).

### Predictor variables

Patient characteristics (Additional file 1: Table S2) were self-reported in the 45 and Up Study baseline questionnaire. Socio-demographic characteristics included age (at questionnaire completion), sex, country of birth, marital status, highest educational qualification, annual household income, working status, private health insurance, and number of people outside home on whom the patient can depend. The total number of positive lifestyle behaviours (ranging from 0 to 4) summed the following: non-smoking; safe level of alcohol consumption (<14 drinks/week); at least 2.5 h of intensity-weighted physical activity per week; and daily consumption of ≥2 serves of fruits and ≥5 serves of vegetables [39, 40]. Health characteristics included Body Mass Index (BMI, based on self-reported height and weight), self-rated general health, psychological distress, number of chronic health conditions, and number of medications taken. Chronic conditions including cancer (excluding skin cancer), heart disease, high blood cholesterol, high blood pressure, diabetes, thrombosis, asthma, depression, anxiety, Parkinson’s disease, thyroid problems and joint/bone problems (osteoarthritis, osteoporosis, low bone density, knee or hip replacement) were defined based on the questions *“Has a doctor ever told you that you have…”; “Age when condition was first found…”; “In the last month have you been treated for ...”;* and *“Have you ever had any of the following operations …”.* Psychological distress was measured by the Kessler-10 scale and categorised as low (total score 10–15), moderate (15–21), high (22–29) and very high (30–50) [41, 42]. The number of medications taken was derived from the items “*Have you taken any medications, vitamins or supplements for most of the last 4 weeks*?”, with check-boxes for up to 25 common medications.

Socio-economic status (SES) of residential areas was based on the Index of Relative Socio-economic Advantage and Disadvantage subscale of the Socio-Economic Indexes for Areas (SEIFA) [43] and grouped into quintiles (quintile 1 indicates lowest SES and quintile 5 indicates highest SES). Remoteness of residential areas was based on Accessibility/Remoteness Index of Australia Plus scores, and classified as major cities (0 < score ≤ 0.2), inner regional (0.2 < score ≤ 2.4), outer regional (2.4 < score ≤ 5.92), remote (5.92 < score ≤ 10.53), and very remote (score > 10.53) [44].

Characteristics of the calls (Table 2) included patient age at call, time of call, caller-patient relationship, original intention, and triage protocols utilised. The “after-hours” period included calls made between 6 pm and 8 am Mondays to Fridays, between 12 pm Saturdays and 8 am Mondays, and on public holidays. The triage protocols were grouped into clinically meaningful categories (see Table 2). The category “Seen by a healthcare provider earlier” indicated patients who rang the healthdirect helpline with a health concern for which they had earlier seen a healthcare provider. In these cases the nurse triages whether the patient’s situation has changed since the last time patient saw their healthcare provider and reviews the advice provided by their health provider in order to assist the patient in managing their health concern.

**Table 2:** Characteristics of 11,088 healthdirect calls, 1 July 2008–15 December 2011 according to dispositions

Call characteristics	Dispositions (N, %^a^)				Total N (%^a^)
	Attend ED immediately	See doctor immediately, in 4 or 24 h	Self-care	Low-urgency^b^	
Patient sex					
Male	698 (42.6%)	2319 (36.3%)	538 (32.5%)	523 (37.1%)	4078 (36.8%)
Female	940 (57.4%)	4065 (63.7%)	1118 (67.5%)	887 (62.9%)	7010 (63.2%)
Patient’s age (at call)					
45–54	344 (21.0%)	1479 (23.2%)	394 (23.8%)	260 (18.4%)	2477 (22.3%)
55–64	494 (30.2%)	1942 (30.4%)	443 (26.8%)	381 (27.0%)	3260 (29.4%)
65–74	430 (26.3%)	1601 (25.1%)	430 (26.0%)	376 (26.7%)	2837 (25.6%)
75+	370 (22.6%)	1362 (21.3%)	389 (23.5%)	393 (27.9%)	2514 (22.7%)
Time of call					
In-hours	378 (23.1%)	1871 (29.3%)	497 (30.0%)	505 (35.8%)	3251 (29.3%)
After-hours	1260 (76.9%)	4513 (70.7%)	1159 (70.0%)	905 (64.2%)	7837 (70.7%)
Caller patient relationship					
Self	1214 (74.1%)	5256 (82.3%)	1444 (87.2%)	1266 (89.8%)	9180 (82.8%)
Spouse, partner	258 (15.8%)	633 (9.9%)	120 (7.2%)	78 (5.5%)	1089 (9.8%)
Other	152 (9.3%)	441 (6.9%)	78 (4.7%)	60 (4.3%)	731 (6.6%)
Unknown	14 (0.9%)	54 (0.8%)	14 (0.8%)	6 (0.4%)	88 (0.8%)
Original intention					
Self-care at home	243 (14.8%)	889 (13.9%)	358 (21.6%)	225 (16.0%)	1715 (15.5%)
Call ambulance or attend ED	519 (31.7%)	1266 (19.8%)	158 (9.5%)	170 (12.1%)	2113 (19.1%)
Contact doctor or healthcare provider	241 (14.7%)	1706 (26.7%)	328 (19.8%)	389 (27.6%)	2664 (24.0%)
Did not know what to do	539 (32.9%)	2125 (33.3%)	661 (39.9%)	500 (35.5%)	3825 (34.5%)
Missing	96 (5.9%)	398 (6.2%)	151 (9.1%)	126 (8.9%)	771 (7.0%)
Triage protocol					
Skin, wound	79 (4.8%)	512 (8.0%)	57 (3.4%)	49 (3.5%)	697 (6.3%)
Limbs and extremities	118 (7.2%)	738 (11.6%)	126 (7.6%)	93 (6.6%)	1075 (9.7%)
Bite, burns, chemical exposure	107 (6.5%)	285 (4.5%)	243 (14.7%)	12 (0.9%)	647 (5.8%)
Respiratory	103 (6.3%)	364 (5.7%)	45 (2.7%)	41 (2.9%)	553 (5.0%)
Head, neck, face non-injury	67 (4.1%)	255 (4.0%)	27 (1.6%)	110 (7.8%)	459 (4.1%)
Neurological, headache, seizure	58 (3.5%)	685 (10.7%)	39 (2.4%)	105 (7.4%)	887 (8.0%)
Abdominal pain or injury	213 (13.0%)	583 (9.1%)	20 (1.2%)	113 (8.0%)	929 (8.4%)
Cold, flu, fever	24 (1.5%)	281 (4.4%)	66 (4.0%)	18 (1.3%)	389 (3.5%)
Postoperative	43 (2.6%)	313 (4.9%)	52 (3.1%)	26 (1.8%)	434 (3.9%)
Cardiac	360 (22.0%)	421 (6.6%)	33 (2.0%)	184 (13.0%)	998 (9.0%)
Bleeding	82 (5.0%)	305 (4.8%)	36 (2.2%)	84 (6.0%)	507 (4.6%)
Nausea, vomiting	52 (3.2%)	182 (2.9%)	63 (3.8%)	17 (1.2%)	314 (2.8%)
Gastrointestinal	67 (4.1%)	316 (4.9%)	79 (4.8%)	203 (14.4%)	665 (6.0%)
Head, neck, face injury	55 (3.4%)	141 (2.2%)	48 (2.9%)	27 (1.9%)	271 (2.4%)
Seen by a provider earlier	0 (0.0%)	293 (4.6%)	180 (10.9%)	88 (6.2%)	562 (5.1%)
Other symptoms	210 (12.8%)	710 (11.1%)	542 (32.7%)	240 (17.0%)	1701 (15.3%)
Total	1638 (100%)	6384 (100%)	1656 (100%)	1410 (100%)	11,088 (100%)

^a^: Percentage of the column

^b^: Including dispositions: see doctor in 72 h or 2 weeks, see a dentist in 72 h, in 2 weeks or when available, see a health provider in 72 h, in 2 weeks or when available

### Statistical analyses

Rates of compliance and self-referral were expressed as percentage with 95% confidence interval (95%CI). Predictors of study outcomes were explored using contingency tables and logistic regression modelling. Both crude odds ratios and adjusted odds ratios (aORs) and 95%CI were computed. The main multivariable models included patient age at call, sex, country of birth, marital status, number of people the patient can depend on, education, household income, working status, private health insurance, SEIFA, positive lifestyle behaviours, BMI, self-rated health status, psychological distress, number of medications taken, time of call, caller-patient relationship, original intention, and triage protocols utilised. Due to collinearity between variables, models examining remoteness excluded the SEIFA variable and models examining number of health conditions excluded the number of medications taken variable. Missing information was treated as a separate category for any variables with missing data (modelling results not shown).

During the study period, 15% of the participants made more than one call to the healthdirect helpline. Sensitivity analysis was conducted, in which two-level multivariable logistic regression analyses were performed to assess the association between outcomes of call and patient and call-related factors. The two-level regression models took into account the clustering of calls within an individual patient. The single-level and two-level analyses yielded similar results (two-level modelling results not shown). SAS version 9.3 was used for descriptive and single-level regression analyses while MLwiN version 2.4 was used for two-level regression models.

## Results

### Data linkage, exclusions and inclusions

The data linkage process identified 17,280 participants in the 45 and Up Study who were the subjects of 26,392 calls to the healthdirect helpline between July 2008 and December 2012. The following categories of calls were excluded from the analysis: calls that did not require triage; missing triage disposition information; inconsistent patient sex and/or age; highly implausible caller-patient relationship given patient’s age; calls made after 15 December 2011 (to ensure at least two weeks of follow-up for MBS claims); and calls that were completed with dispositions other than those included in the defined study outcomes [45]. In addition, Department of Veterans’ Affairs (DVA) participants (*n* = 268) were excluded because their primary care is subsidised through the DVA and therefore not captured in MBS data. When a patient had more than one call in a day, only the last call was selected for the analyses. Following exclusion of 15,304 calls relating to 8874 patients, the final healthdirect helpline dataset included 11,088 calls made between 1 July 2008 and 15 December 2011, relating to 8406 patients. The included and excluded subjects of call had similar socio-demographic, lifestyle and health characteristics (comparison results not shown).

### Patient characteristics

Among the 8406 patients, 61.5% were female, 66.6% were living with a partner, and 23.6% were born overseas. In terms of educational qualifications, 32.5% had a school certificate, 31.8% had a trade, apprenticeship or diploma certificate and 18.1% had a university or higher degree. Nearly a quarter (23.5%) reported annual family income of AUD50,000 or higher, 38.6% were working and 53.8% had private health insurance. About 28.9% of patients lived in socio-economically disadvantaged areas (SEIFA quintile 1 and 2), while 41.5% lived in inner regional and 18.3% in remote and very remote areas of NSW (Additional file 1: Table S2).

In terms of healthy lifestyle behaviours, 281 patients (3.3%) did not report any positive behaviours while 1672 (19.9%), 3145 (37.4%) and 3308 (39.4%) patients respectively reported one, two, and three or four positive behaviours. There were 2019 (24.0%) patients with a BMI ≥ 30.0, and 1824 patients (21.7%) rated their general health as fair or poor. The proportion of patients reporting high blood pressure and high cholesterol was 41.0% and 29.8% respectively, while depression (23.6%) and joint/bone problems (21.7%) were also common. Other chronic health conditions included heart disease (17.9%), asthma (17.9%), anxiety (16.9%), cancer (13.7%), diabetes (12.5%), thrombosis (7.1%), thyroid problems (6.7%) and Parkinson’s disease (1.0%). More than half of patients (57.6%, *n* = 4838) had two or more conditions and 40.3% (*n* = 3389) reported taking two or more medications. In addition, 1007 (12.0%) participants reported high or very high levels of psychological distress (Additional file 1: Table S2).

### Call characteristics

Among 11,088 calls included in the analysis (Table 2), 1638 (14.8%) were given the disposition “Attend ED immediately”, 6384 (57.6%) were given the dispositions “See a doctor immediately, in 4 hours or 24 hours”, 1656 (14.9%) were advised to self-care at home, and 1410 (12.7%) were advised to seek care in a less urgent timeframe. The mean age of the patients when they contacted the healthdirect helpline was 64.8 years (±11.5), 63.2% of the calls were for female patients, 82.8% were made by the patients themselves and 70.7% of calls were made after-hours.

Calls concerning symptoms of limbs and extremities and symptoms of the cardiac system accounted for 9.7% and 9.0% respectively of total calls, while abdominal pain or injury, and neurological symptoms, headache or seizures each accounted for more than 8% of total calls. The protocol “Seen by a healthcare provider earlier” was used for 5.1% of the calls, none of which ended with advice to attend ED immediately (Table 2). When asked about their intention prior to calling the healthdirect helpline, 31.7% of patients given the advice “Attend ED immediately” stated that their intention had been to contact ambulance services or go to ED (Table 2).

### Rates of compliance and self-referral

As presented in Table 3, compliance with the advice “Attend ED immediately” was 68.6% (95%CI 66.4–70.9), with the advice “See a doctor immediately, in 4 hours or 24 hours” was 64.6% (95%CI 63.4–65.8) and with advice to “Self-care” was 77.5% (95%CI 75.5–79.5). Of 3066 patients who were given self-care and low-urgency dispositions, 7.0% (95% CI 6.1–7.9) self-referred to ED (*n* = 189) or hospital (*n* = 26) within 24 h of the call.

**Table 3:** Rates (95%CI) of compliance and self-referral to urgent care, and use of health services

	Compliance with triage advice			Self-referral to ED or hospital in 24 hours^c^
	Attend ED immediately	See doctor immediately, in 4 hours or 24 h	Self-care	
Total number of calls	1638	6384	1656	3066
Number (compliance or self-referral)	1124	4123	1284	215
Rate (95%CI),%	68.6 (66.4–70.9)	64.6 (63.4–65.8)	77.5 (75.5–79.5)	7.0 (6.1–7.9)
Use of health services				
ED in 24 h	1084	2038	75	189
ED in 25–48 h	13	48	20	26
ED >48 h	26	99	45	75
Hospital in 24 hours^a^	40	64	17	26
Hospital in 25–48 hours^a^	0	9	5	7
Hospital >48 hours^a^	8	52	22	44
MBS on the same day	77	1352	101	247
MBS in the next day	75	670	154	349
MBS in day 3 or later	282	1800	1067	1834
No linked record ^b^	33	252	150	269

^a^: There is no preceding ED record

^b^ No linked record of ED visit, hospital admission or MBS claim until the next call

^c^: Dispositions include self-care, see doctor in 72 h or 2 weeks, and see a dentist or a health provider in 72 h, 2 weeks or when available

### Factors associated with compliance and self-referral

Table 4 and Additional file 1: Table S3 present adjusted odds ratios for compliance and self-referral according to patient and call-related factors. Among socio-demographic characteristics, compliance and self-referral were most influenced by the patient’s age, working status, SES and remoteness of residence. Compared to patients aged 45–54 years, patients aged ≥55 years were more likely to comply with advice to see a doctor but were less likely to self-care as advised. Higher likelihood of compliance with advice to see a doctor was found among patients who worked full time. Compared to patients living in SEIFA quintile 1 (lowest SES), those living in higher SES quintile areas were more likely to follow the advice to attend ED (Quintile 3), or to see a doctor (Quintile 3, Quintile 4) while being less likely to self-refer to ED/hospital (Quintile 5). Compared to those living in major city areas, patients living in rural and remote areas had significantly lower compliance with advice to attend ED (aOR = 0.67, 95%CI 0.49–0.92), and to see a doctor (aOR = 0.59, 95%CI 0.51–0.69) while compliance with self-care advice was not significantly different (aOR = 1.33, 95%CI 0.91–1.94).

**Table 4:**
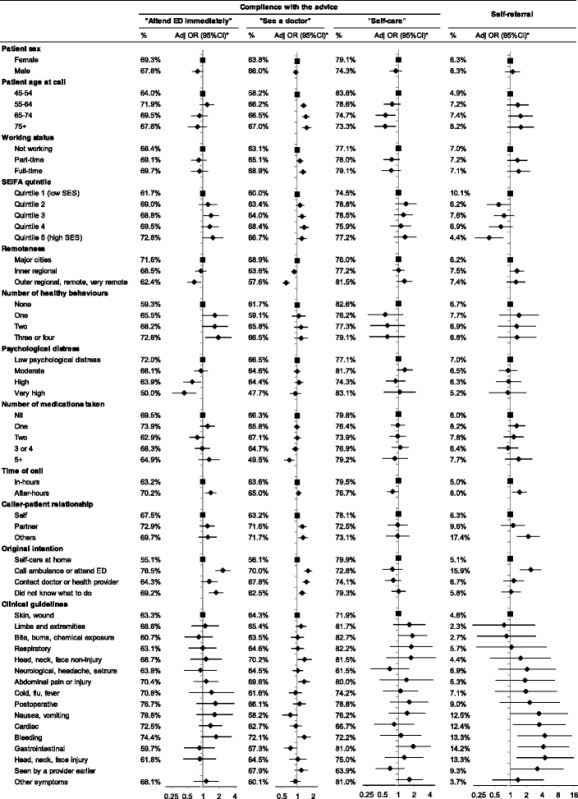
Compliance and self-referral according to patient and call characteristics, % and adjusted odds ratio (95%CI)

Adjusted for patient age at call, sex, country of birth, marital status, people can depend on, education, household income, working status, private health insurance, SEIFA, healthy behaviours, BMI, general health, medication taken, time of call, caller-patient relationship, original intention, and triage protocols. Models examining remoteness did not include SEIFA, models examining morbidity did not include number of medications taken. See Additional file 1: Table S3 for further details. Forest plots: The diamond shapes represent adjusted odds ratios, the horizontal bars represent the 95%CIs, the square shapes represent reference categories.

Compared to patients who reported no healthy behaviours, those with three or four positive behaviours were more likely to follow the advice to attend ED immediately (aOR = 2.09, 95%CI 1.13–3.87). Patients reporting high or very high levels of psychological distress were significantly less likely to attend ED as advised (aOR = 0.61, 95% CI 0.40–0.92 and aOR = 0.42, 95% CI 0.24–0.74, respectively) compared to those having low level of psychological distress. The likelihood of following advice to see a doctor was lower among those who were taking five or more medicines.

Calls made after-hours were associated with both higher compliance with advice to attend ED and higher self-referral to ED. Compliance with advice to see a doctor was more likely among calls made by a partner or spouse, or by a carer, parent or grandchild, than among patients calling on their own behalf. Compared to callers whose original intention was to self-care, those who had originally intended to contact ambulance services or attend ED were significantly more likely to present to ED as advised (aOR = 2.59, 95%CI 1.82–3.69), see a doctor as advised (aOR = 1.69, 95%CI 1.40–2.03) and to present to ED contrary to advice (aOR = 2.64, 95%CI 1.59–4.38). Patients who originally intended to contact a doctor or a health provider were more likely to follow the advice to see a doctor (aOR = 1.56, 95%CI 1.31–1.86).

Particular patient symptoms were significantly associated with patient self-referral to ED. Compared to patients with skin or wound problems, self-referral was significantly higher among those with cardiac symptoms (aOR = 3.94, 95%CI 1.14–13.63), bleeding (aOR = 5.15, 95%CI 1.41–18.75), gastrointestinal problems (aOR = 4.81, 95%CI 1.42–16.28) and head, neck and face injuries (aOR = 5.17, 95%CI 1.33–20.04), as well as among those who had seen a provider earlier (aOR = 3.60, 95%CI 1.04–12.51) (Table 4 and Additional file 1: Table S3).

## Discussion

This is the first research study to comprehensively evaluate the utilisation of ED, hospital and primary care services among middle-aged and older patients, following consultation with the Australian healthdirect helpline. To our knowledge, it is the only study internationally to date that has linked records of triage calls to detailed self-reported information about patients’ socio-demographic and health characteristics and major administrative data collections.

Rates of compliance in this study are in line with those reported in the international literature [9, 10], including compliance with advice to “attend ED” (68.6% in this study vs reported median 75%), to attend office-care (64.6% in this study vs reported median 66%) and for self-care (77.5% vs reported median 77%). Compared with a previous study, which also examined healthdirect helpline calls but only among people who lived within 2 km from Fremantle Hospital in Western Australia [11], our study found a lower rate of self-referral to acute care services (7.0%, 95%CI 6.1–7.9 vs 9.0%, 95%CI 8.0–10.0). Compared with De Coster’s Canadian study [29] which used a similar data linkage method, patients calling the healthdirect helpline had higher rates of compliance with advice to attend ED (68.6% vs 52.3%) and to see a doctor (64.6% vs 43.2%), and lower compliance with self-care advice (77.5% vs 83.7%). Discrepancies in compliance rates between the two studies are likely due to differences in patient age, quality of record linkage, and completeness of claim data. In De Coster’s study [29], patients under four years old accounted for 43% of study participants, while patients aged 50 years and older accounted for only 8.6%. Previous meta-analyses showed that compliance with advice to seek ED care or self-care is higher among parents of paediatric patients than among adult patients [10]. The probabilistic linkage of our data yielded extremely low rates of missed links (0.43% vs 22% in the Canadian study) [29, 37]. The MBS data in our study (deterministically linked) included all GP visits financed by the Medicare program [34], while De Coster and colleagues mentioned that some visits to physicians who did not bill for their services might have been missed [29].

Although an extensive body of research has investigated patient compliance with treatment regimens (30%–80%) [46–49], findings have been inconsistent [46–48]. This is perhaps partly due to heterogeneity of populations studied and variations and challenges in measuring compliance. According to reviews [46–49], patient compliance tends to increase with older age, higher levels of understanding about the disease and treatment, and higher levels of social support, while gender, education, ethnicity, income and marital status have not shown to be consistent predictors of compliance. The current study found significant variations according to patient’s age in compliance with advice to see a doctor and self-care but not in compliance to attend ED or self-referral to ED. This indicates clinical acuity has a mediating influence on the relationship between patient’s age and compliance with the triage advice. Level of social support (i.e. living with a partner and the number of people on whom the patient can depend) was not associated with compliance or self-referral. Similarly, consistent with results of the Canadian data linkage study [29], patient’s socio-economic characteristics were not associated with the study outcomes. The role of neighbourhood contexts over and above the effects of individual socio-economic position [50, 51], however, is highlighted in this study. Lower compliance with advice to attend ED or see a doctor among rural and remote patients may reflect barriers to service utilisation in those areas, such as greater travelling distance, lack of availability and accessibility of services, and greater variability in service availability in the after-hours period. [52]. It should be noted that this study may have underestimated compliance in rural and remote areas, as some patients may have sought care in neighbouring States/Territories for which this study did not have data, or attended small remote hospitals that are not included in the ED dataset.

Prior studies reported that compliance with therapies is often lower in patients with risky behaviours (tobacco smoking, high alcohol intake, and illicit drug use) [46]. In this study, patients had greater compliance with ED-care advice when they displayed three or more positive health behaviours when compared with those with no health behaviours, but there were no effects on other outcomes of the call. In contrast to the Canadian study [29], this research found little impact of patient health characteristics on compliance or self-referral, with the exception of lower compliance with ED-care advice among people with high and very high levels of psychological distress. The self-reported health conditions examined in this study were mainly chronic; they did not necessarily reflect the circumstances and acuity of the presenting symptoms at the time of call. Among calls triaged to seek ED care, patients with very high psychological distress were more likely than other patients to seek advice for acute health reasons, including diabetes out of control (19.4% vs <7.0%) and asthma attack (7.4% vs <2.0%). Patients with a high level of psychological distress also had higher proportions of calls relating to acute alcohol intoxication (7.1% vs <1% in other patients). These results highlight the difficulties in triaging health symptoms in the presence of comorbid psychological distress, and the need for triage staff to consider the appropriateness of the reached disposition in the clinical context of the individual patient and their reported symptoms before completing the interaction.

This study found little variation in compliance (attend ED, see a doctor, and self-care) according to triage protocols, which was contrary to the Canadian data linkage study [29]. It should be noted, however, that the Canadian study included patients of all ages (43% were children younger than four years of age) and the application of the necessarily broader triage protocols reduces comparability with the current study of patients ≥45 years. Our study was able to distinguish clinical symptoms associated with patient self-referral to ED. The rate of self-referral was significantly higher following calls concerning cardiac symptoms, bleeding, gastrointestinal problems, and head injuries. Further, time to arrival at ED among patients with these symptoms was shorter than those reporting other symptoms. The percentage of patients self-referring to ED within 4 h was 73.7% among those with bleeding, 70.0% among those with head, neck and face injuries, and 70.0% among those with cardiac symptoms, compared to approximately 55% among patients with postoperative concerns or nausea and vomiting. Higher self–referral and earlier presentations at ED would not be unexpected given the likelihood that these symptoms might cause patient anxiety and progress rapidly.

The results of the current study provide insight into the caller’s original intention and subsequent behaviours. Any interpretation must be made in recognition of the fact that, it is not always practical for healthdirect triage nurses to collect the caller’s “original intention” at the beginning of the triage, it may have been influenced by a number of factors, including the advice given, the process of the call, or the caller’s satisfaction with the interaction with the staff. Earlier studies [14, 15] that asked the caller’s original intention prior to the triage process showed reasonably high levels of compliance with advice despite discrepancies between their original intention and the advice received [14, 15]. In the current study, compliance with advice to attend ED or see a doctor was greater when the triage advice matched the patient’s original intention, but this pattern was not seen among the self-care group. This study, for the first time, investigated the relationship between patient intention and self-referral to acute care. After controlling for the effect of after-hours time of call, patients intending to seek urgent care (ambulance or ED) were significantly more likely to present to ED or hospital. This warrants further studies among patients triaged to low-urgency care and suggests that where there is a large discrepancy between the triage advice and the caller’s original intention, reassurance may be needed to encourage compliance with low-urgency disposition. This also highlights the importance in discussing with patients their ability to access the appropriate level of care within the advised time frame. A previous study of patients ≥70 years who presented to ED for non-urgent health problems [53] found that the patients had difficulties in accessing primary care and they often perceived ED care as a specialised service. However, in this study, the possibility of symptom evolution or progression cannot be ruled out, particularly as self-referred patients presented significantly later than those advised to attend ED (38.6% vs 90.2% attended within 2 h).

### Limitations

While this study is unique in using detailed, self-reported patient information linked to comprehensive administrative data sources, some limitations exist. Records of non-admitted attendances at small rural EDs may have been missed, although these presentations account for only around 4% of all ED presentations in NSW [36]. The study was unable to identify patients who attended ED or hospital in other States/Territories (except one hospital in the ACT). Furthermore, participants in the 45 and Up Study may be more “health conscious” than the general population [33, 54, 55] and may be more likely to seek health care and follow given advice. However, internal relative risk estimates from the 45 and Up Study for a wide range of exposure-outcome relationships are comparable with those from population health surveys [54].

### Implications for practice

Telephone triage services such as the healthdirect helpline direct patients to the services that best suit their reported health symptoms. Older patients, with their rising numbers, higher demand for emergency and complex care, greater risk of symptom and disease deterioration, and poorer health care outcomes, are a prominent and growing client group of these services. Non-compliance with advice to attend ED care has the potential for serious adverse health outcomes for patients. The lower compliance rates in rural and remote areas highlights the need for triage staff, when interacting with callers from these areas to discuss options that best suits the patient’s circumstances. Incorporating extra targeted questions for patients who indicate an original intention to seek care of a higher intensity than the triage advice may assist in identifying important information not elicited or discussed during the triage process. In addition, during the triage if the patient shows signs of distress or anxiety, triage staff could be advised to place greater emphasis on building a good verbal rapport with the patient and encourage patient to follow the advice.

## Conclusions

Compliance with telephone triage advice among middle-aged and older patients varied substantially according to both patient- and call-related factors. Knowledge about the patients who are less likely to comply with telephone triage advice, and about characteristics of calls that may influence compliance, will assist in refining patient triage protocols and referral pathways, training staff and and tailoring service design and delivery to achieve optimal patient compliance.

## Additional file

Additional file 1: Table S1. Algorithm to define the timing of the MBS claims. **Table S2.** Socio-demographic, lifestyle and health characteristics of 8406 subjects of calls. **Table S3.** Compliance and self-referral according to patients and call characteristics, number (%), crude and adjusted odds ratio (95%CI). (DOCX 53 kb)

## Abbreviations

ACT: Australian Capital Territory
DVA: Department of Veterans’ Affairs
ED: emergency department
GP: general practitioner
MBS: Medicare Benefits Schedule
NSW: New South Wales
SEIFA: Socio-Economic Indexes for Areas
SES: socio-economic status.
