# Supplementary material for: Compliance with telephone triage advice among adults aged 45 years and older: an Australian data linkage study
Source: BMC Health Serv Res. 2017 Aug 1;17:512. doi: 10.1186/s12913-017-2458-y (PMC5539620; doi:10.1186/s12913-017-2458-y)
Supplement: Additional file 1: Table S1. — Algorithm to define the timing of the MBS claims. Table S2. Socio-demographic, lifestyle and health characteristics of 8406 subjects of calls. Table S3. Compliance and self-referral according to patients and call characteristics, number (%), crude and adjusted odds ratio (95%CI). (DOCX 53 kb) [file 12913_2017_2458_MOESM1_ESM.docx]

**Supplement Table S1: Algorithm to define the timing of the MBS claims**

The algorithm aimed to assign the timing of the medical consultation relative to the time of call and time of arrival or discharge from ED/hospital (where applicable), taking into account the following factors:

- Presence of a MBS claim and/or ED/hospital visit on the same day of call (day 1), day after the call (day 2), and day 3 and later;
- Time of calls: business-hours, after-hours, exact time of call. Calls made between 6PM and 8AM Mondays to Fridays, between 12PM Saturdays and 8AM Mondays, and on public holidays were categorised as after-hours.
- Type of MBS items: as after-hours specific or generic times. Definition of after-hours consultations was the same as the definition for after-hours calls.
- Time of arrival at ED/hospital admission, time of discharge from ED/hospital; and
- Constant parameters: duration of call assumed to be 10 minutes, duration of consultation assumed to be 20 minutes, and time to arrival at the ED/hospital following the consultation assumed to be 20 minutes.

| **Type of MBS items** | **Time of call** | **ED/hospital** | **Assigned timing of the consultation** | **Rationale and Comments** |
| --- | --- | --- | --- | --- |
| **On day 1 (day of the call): Presence of MBS claim and absence of ED/hospital** | | | | |
| Any items | Business-hours | n/a | After the call | Most of these calls were between 8AM and 12PM, which would allow adequate amount of time to see a doctor following the call. |
| After-hours specific | After-hours | n/a | After the call | Most of these calls were between 8AM and 8PM, patients may have attended practices which opened extended hours. |
| Generic items | After-hours, before 8AM | n/a | After the call |  |
| Generic items | After-hours, between 8AM-6PM | n/a | After the call | It is assumed that the helpline call was the first point of contact for the presenting symptoms. Patients may have attended practices which opened extended hours. Less than 30 calls have these characteristics. |
| Generic items | After-hours, after 6PM | n/a | Before the call | Most of these calls were after 9PM. About 40% were told to seek care immediately or within 4 hours. Patients might have seen a doctor earlier and experienced deterioration. About 50% were told to self-care or seek care with timeframe ≥24 hours, these patients might have seen a doctor earlier and have contacted healthdirect for non-urgent concerns. |
| **On day 1 (day of the call): Presence of both MBS claim and ED/hospital** | | | | |
| Any items | Business-hours | - Overnight stay at ED/hospital or discharge after 9PM | After the call and before ED/ hospital arrival |  |
|  |  | - Discharge before 9PM and computed time from call to ED/hospital >50 minutes^§^ | After the call and before ED/ hospital arrival | Patient is assumed to have enough time to see a doctor who then referred patient to the ED |
|  |  | - Discharge before 9PM and computed time from call to ED/hospital <50 minutes^§^ | - After the call if disposition was to see a GP/doctor. - After discharge from ED/hospital if disposition was to attend ED. | Patient is assumed to follow triage advice. Less than 20 calls have these characteristics. |
| After-hours specific | After-hours | - Overnight stay at ED/hospital or discharge after 9PM | After the call and before ED/ hospital arrival |  |
|  |  | - Discharge before 9PM and computed time from call to ED/hospital >50 minutes^§^ | After the call and before ED/ hospital arrival | Patient is assumed to have enough time to see a doctor who then referred patient to the ED. |
|  |  | - Discharge before 9PM and computed time from call to ED/hospital <50 minutes^§^ | - After the call if disposition was to see a GP/doctor. - After discharge from ED/hospital if disposition was to attend ED. | Patient is assumed to follow triage advice. Less than 20 calls have these characteristics. |
| Generic items | After-hours, before 8AM | - Computed time from call to ED/hospital ≤50 minutes^§^ | After discharge from ED/hospital | Most patients in this category had immediate ED/hospital visit after the call and were discharge before 3PM. Patient is assumed to follow the ED/hospital discharge instructions to attend a GP. |
|  |  | - Computed time from call to ED/hospital >50 minutes^§^ | After the call and before ED/ hospital arrival | Patient is assumed to have enough time to see a doctor following the call. Most patients in this category presented to ED/hospital >4hours after the call and either stayed overnight or were discharged after 9PM. |
| Generic items | After-hours, between 8AM and 3PM | Any | After discharge from ED/hospital | Less than 10 calls have these characteristics. Patients had ED/hospital visit immediately after the call and were discharged before 3PM. It is assumed that the patient followed the discharge instructions to attend a GP. |
| Generic items | After-hours, between 3PM and 6PM | Any | Before the call | Less than 10 calls have these characteristics. Patients had ED/hospital visit immediately after the call and stayed overnight at ED/hospital. Patients might have experienced deterioration. |
| Generic items | After-hours, after 6PM | Any | Before the call | Most patients had ED/hospital visit immediately after the call and stayed overnight at ED/hospital. Patients might have seen a doctor earlier and experienced deterioration. |
| **On day 2 (the next day): Presence of both MBS claim and ED/hospital** | | | | |
| Any | Any | Time of arrival at ED/hospital before 10 AM of the next day | After discharge from ED/hospital | Most of these patients called around midnight, had immediate ED/hospital visit after the call, had short stay at the ED/hospital, and were discharged before 12PM. Patient is assumed to follow the discharge instructions to attend a GP. |
| Any | Any | Time of arrival at ED/hospital after 10 AM of the next day | After the call and before ED/ hospital arrival | Most of these patients stayed overnight at ED/hospital. |
| **On day 3 and later: Presence of both MBS claim and ED/hospital** | | | | |
| Any | Any | Any | After the call and before ED/ hospital arrival | Most of the patients arrived at ED/hospital after 11AM and either stayed overnight at ED/hospital or were discharged after 9PM. |
| ^§^: Given constant parameters: 10 minutes duration of call, 20 minutes duration of consultation, and 20 minutes to arrival at ED/hospital  n/a: not applicable | | | | |

**Supplement Table S2: Socio-demographic, lifestyle and health characteristics of 8406 subjects of calls**

| **Patient characteristics** | | **Number** | **Percentage** |
| --- | --- | --- | --- |
| **Socio-demographic** | |  |  |
| Sex |  |  |  |
|  | Male | 3238 | 38.5% |
|  | Female | 5168 | 61.5% |
| Age (at questionnaire completion) | |  |  |
|  | 45-54 | 2221 | 26.4% |
|  | 55-64 | 2573 | 30.6% |
|  | 65-74 | 2024 | 24.1% |
|  | 75+ | 1588 | 18.9% |
| Country of birth | |  |  |
|  | Australia | 6421 | 76.4% |
|  | Other | 1985 | 23.6% |
| Marital status | |  |  |
|  | Without a partner | 2747 | 32.7% |
|  | With a partner | 5598 | 66.6% |
|  | Unknown | 61 | 0.7% |
| Number of people can depend on | |  |  |
|  | No one | 682 | 8.1% |
|  | 1-2 people | 1758 | 20.9% |
|  | 3-4 people | 1730 | 20.6% |
|  | 5-7 people | 1682 | 20.0% |
|  | ≥8 people | 2167 | 25.8% |
|  | Unknown | 387 | 4.6% |
| Education | |  |  |
|  | No school certificate or other qualification | 1328 | 15.8% |
|  | School or intermediate certificate | 1994 | 23.7% |
|  | Higher school or leaving certificate | 739 | 8.8% |
|  | Trade or apprenticeship | 947 | 11.3% |
|  | Certificate or diploma | 1720 | 20.5% |
|  | University degree or higher | 1522 | 18.1% |
|  | Unknown | 156 | 1.9% |
| Annual household income (AUD) | |  |  |
|  | Less than $30,000 | 3371 | 40.1% |
|  | $30,000-$49,999 | 1257 | 15.0% |
|  | $50,000-$69,999 | 732 | 8.7% |
|  | $70,000 or more | 1244 | 14.8% |
|  | Won’t disclose | 1296 | 15.4% |
|  | Missing | 506 | 6.0% |
| Working status | |  |  |
|  | Not working | 4984 | 59.3% |
|  | Part time | 1529 | 18.2% |
|  | Full time | 1714 | 20.4% |
|  | Unknown | 179 | 2.1% |
| Private health insurance | |  |  |
|  | Private health insurance | 4520 | 53.8% |
|  | Concession card | 2347 | 27.9% |
|  | None of these | 1343 | 16.0% |
|  | Unknown | 196 | 2.3% |
| SEIFA Quintile | |  |  |
|  | Quintile 1 (lower SES) | 864 | 10.3% |
|  | Quintile 2 | 1564 | 18.6% |
|  | Quintile 3 | 3355 | 39.9% |
|  | Quintile 4 | 1610 | 19.2% |
|  | Quintile 5 (higher SES) | 1013 | 12.1% |
| Remoteness | |  |  |
|  | Major cities | 3376 | 40.2% |
|  | Inner regional | 3488 | 41.5% |
|  | Outer regional, remote, very remote | 1542 | 18.3% |
| **Lifestyle and health characteristics** | |  |  |
| Positive health behaviours | |  |  |
|  | None | 281 | 3.3% |
|  | One | 1672 | 19.9% |
|  | Two | 3145 | 37.4% |
|  | Three or fore | 3308 | 39.4% |
| Body Mass Index (BMI kg/m^2^) | |  |  |
|  | Under-weight (BMI<18.5) | 124 | 1.5% |
|  | Normal weight (18.5≤BMI<25.0) | 2697 | 32.1% |
|  | Overweight (25.0≤BMI<30.0) | 2848 | 33.9% |
|  | Obese (30.0≤BMI) | 2019 | 24.0% |
|  | Missing | 718 | 8.5% |
| Self-rated general health | |  |  |
|  | Excellent | 849 | 10.1% |
|  | Very good | 2509 | 29.8% |
|  | Good | 2884 | 34.3% |
|  | Fair | 1441 | 17.1% |
|  | Poor | 383 | 4.6% |
|  | Missing | 340 | 4.0% |
| Health conditions | |  |  |
|  | High blood pressure | 3445 | 41.0% |
|  | High blood cholesterol | 2501 | 29.8% |
|  | Depression | 1982 | 23.6% |
|  | Joint/bone problems | 1820 | 21.7% |
|  | Heart disease | 1507 | 17.9% |
|  | Asthma | 1508 | 17.9% |
|  | Anxiety | 1418 | 16.9% |
|  | Cancer (exclude skin cancer) | 1148 | 13.7% |
|  | Diabetes | 1047 | 12.5% |
|  | Thrombosis | 601 | 7.1% |
|  | Thyroid problems | 562 | 6.7% |
|  | Parkinson’s disease | 80 | 1.0% |
| Number of above health conditions | |  |  |
|  | Nil condition | 1550 | 18.4% |
|  | One | 2018 | 24.0% |
|  | Two | 1842 | 21.9% |
|  | 3 or 4 | 2232 | 26.6% |
|  | 5 or more | 764 | 9.1% |
| Psychological distress | |  |  |
|  | Low psychological distress | 4790 | 57.0% |
|  | Moderate | 1444 | 17.2% |
|  | High | 678 | 8.1% |
|  | Very high | 329 | 3.9% |
|  | Unknown | 1165 | 13.9% |
| Number of medications taken | |  |  |
|  | Nil medications | 3057 | 36.4% |
|  | One | 1960 | 23.3% |
|  | Two | 1420 | 16.9% |
|  | 3 or 4 | 1509 | 18.0% |
|  | 5 or more | 460 | 5.5% |
|  | | | |

**Supplement Table S3: Compliance and self-referral according to patients and call characteristics, number (%), crude and adjusted odds ratio (95%CI)**

|  | | **Compliance with disposition "Attend ED immediately"** | | |  | **Compliance with disposition "See doctor immediately, in 4 or 24 hours"** | | |  | **Compliance with disposition "Self-care"** | | |  | **Self-referral to ED / hospital within 24 hours** | | |
| --- | --- | --- | --- | --- | --- | --- | --- | --- | --- | --- | --- | --- | --- | --- | --- | --- |
|  |  | N (%) | Crude OR (95%CI) | Adjusted OR (95%CI)* |  | N (%) | Crude OR (95%CI) | Adjusted OR (95%CI)* |  | N (%) | Crude OR (95%CI) | Adjusted OR (95%CI)* |  | N (%) | Crude OR (95%CI) | Adjusted OR (95%CI)* |
| **Patient sex** | |  |  |  |  |  |  |  |  |  |  |  |  |  |  |  |
|  | Female | 651 (69.3%) | 1 | 1 |  | 2592 (63.8%) | 1 | 1 |  | 884 (79.1%) | 1 | 1 |  | 127 (6.3%) | 1 | 1 |
|  | Male | 473 (67.8%) | 0.93 (0.76-1.15) | 0.78 (0.60-1.01) |  | 1531 (66.0%) | 1.10 (0.99-1.23) | 0.94 (0.83-1.07) |  | 400 (74.3%) | 0.77 (0.60-0.98) | 0.88 (0.65-1.20) |  | 88 (8.3%) | 1.34 (1.01-1.77) | 1.12 (0.79-1.59) |
| **Patient age at call** | |  |  |  |  |  |  |  |  |  |  |  |  |  |  |  |
|  | 45-54 | 220 (64.0%) | 1 | 1 |  | 861 (58.2%) | 1 | 1 |  | 330 (83.8%) | 1 | 1 |  | 32 (4.9%) | 1 | 1 |
|  | 55-64 | 355 (71.9%) | 1.44 (1.07-1.93) | 1.23 (0.88-1.71) |  | 1285 (66.2%) | 1.40 (1.22-1.61) | 1.36 (1.17-1.58) |  | 348 (78.6%) | 0.71 (0.50-1.01) | 0.74 (0.50-1.10) |  | 59 (7.2%) | 1.50 (0.96-2.33) | 1.43 (0.87-2.37) |
|  | 65-74 | 299 (69.5%) | 1.29 (0.95-1.74) | 0.86 (0.58-1.26) |  | 1065 (66.5%) | 1.43 (1.23-1.65) | 1.52 (1.26-1.83) |  | 321 (74.7%) | 0.57 (0.40-0.81) | 0.54 (0.34-0.86) |  | 60 (7.4%) | 1.56 (1.00-2.43) | 1.63 (0.92-2.88) |
|  | 75+ | 250 (67.6%) | 1.17 (0.86-1.60) | 0.78 (0.50-1.20) |  | 912 (67.0%) | 1.45 (1.25-1.70) | 1.57 (1.27-1.93) |  | 285 (73.3%) | 0.53 (0.37-0.75) | 0.53 (0.32-0.88) |  | 64 (8.2%) | 1.73 (1.12-2.68) | 1.66 (0.90-3.09) |
| **Country of birth** | |  |  |  |  |  |  |  |  |  |  |  |  |  |  |  |
|  | Overseas | 246 (70.9%) | 1 | 1 |  | 942 (65.1%) | 1 | 1 |  | 310 (74.0%) | 1 | 1 |  | 52 (7.0%) | 1 | 1 |
|  | Australia | 878 (68.0%) | 1.15 (0.88-1.49) | 1.12 (0.84-1.50) |  | 3181 (64.4%) | 1.03 (0.91-1.17) | 0.95 (0.83-1.09) |  | 974 (78.7%) | 0.77 (0.59-0.99) | 0.83 (0.63-1.11) |  | 163 (7.0%) | 0.99 (0.71-1.37) | 0.84 (0.59-1.20) |
| **Marital status** | |  |  |  |  |  |  |  |  |  |  |  |  |  |  |  |
|  | Without a partner | 373 (65.0%) | 1 | 1 |  | 1371 (60.4%) | 1 | 1 |  | 516 (76.9%) | 1 | 1 |  | 88 (7.0%) | 1 | 1 |
|  | With a partner | 740 (70.4%) | 1.28 (1.03-1.59) | 1.10 (0.84-1.44) |  | 2730 (67.1%) | 1.34 (1.20-1.49) | 1.13 (1.00-1.28) |  | 757 (78.0%) | 1.07 (0.84-1.35) | 0.99 (0.74-1.31) |  | 126 (7.1%) | 1.01 (0.76-1.33) | 0.97 (0.70-1.35) |
| **People can depend on** | |  |  |  |  |  |  |  |  |  |  |  |  |  |  |  |
|  | No one | 96 (67.6%) | 1 | 1 |  | 338 (61.9%) | 1 | 1 |  | 99 (75.0%) | 1 | 1 |  | 14 (5.6%) | 1 | 1 |
|  | 1 or 2 | 239 (69.1%) | 1.07 (0.70-1.63) | 0.91 (0.58-1.45) |  | 837 (61.9%) | 1.00 (0.81-1.22) | 0.97 (0.78-1.19) |  | 281 (78.5%) | 1.22 (0.76-1.94) | 1.20 (0.72-2.00) |  | 46 (6.8%) | 1.23 (0.67-2.29) | 1.22 (0.63-2.35) |
|  | 3 or 4 | 225 (63.9%) | 0.85 (0.56-1.28) | 0.72 (0.45-1.14) |  | 888 (62.5%) | 1.03 (0.84-1.26) | 1.14 (0.92-1.42) |  | 303 (75.2%) | 1.01 (0.64-1.59) | 0.77 (0.46-1.28) |  | 65 (8.7%) | 1.60 (0.88-2.91) | 1.72 (0.90-3.29) |
|  | 5 to 7 | 233 (72.4%) | 1.25 (0.82-1.92) | 0.93 (0.58-1.51) |  | 803 (66.3%) | 1.21 (0.98-1.49) | 1.09 (0.88-1.36) |  | 240 (78.7%) | 1.23 (0.76-1.99) | 1.09 (0.64-1.85) |  | 32 (5.8%) | 1.04 (0.55-1.99) | 1.10 (0.55-2.20) |
|  | 8+ | 283 (71.6%) | 1.21 (0.80-1.83) | 0.87 (0.55-1.40) |  | 1062 (67.6%) | 1.28 (1.05-1.57) | 1.10 (0.89-1.36) |  | 307 (79.7%) | 1.31 (0.82-2.09) | 1.19 (0.71-2.01) |  | 45 (6.4%) | 1.15 (0.62-2.13) | 1.08 (0.56-2.10) |
| **Education** | |  |  |  |  |  |  |  |  |  |  |  |  |  |  |  |
|  | < School certificate | 180 (67.2%) | 1 | 1 |  | 655 (64.9%) | 1 | 1 |  | 188 (79.3%) | 1 | 1 |  | 34 (7.4%) | 1 | 1 |
|  | School or intermediate certificate | 253 (66.4%) | 0.97 (0.69-1.35) | 0.86 (0.60-1.25) |  | 961 (65.4%) | 1.02 (0.87-1.21) | 0.94 (0.79-1.12) |  | 278 (77.2%) | 0.88 (0.59-1.32) | 0.77 (0.50-1.18) |  | 43 (6.4%) | 0.86 (0.54-1.37) | 0.83 (0.50-1.36) |
|  | Higher school or leaving certificate | 111 (72.1%) | 1.26 (0.82-1.95) | 1.15 (0.71-1.86) |  | 328 (63.9%) | 0.96 (0.77-1.20) | 0.91 (0.72-1.14) |  | 129 (75.9%) | 0.82 (0.51-1.31) | 0.72 (0.43-1.20) |  | 24 (8.3%) | 1.13 (0.66-1.96) | 1.52 (0.84-2.75) |
|  | Trade or apprenticeship | 139 (72.8%) | 1.31 (0.87-1.97) | 1.33 (0.85-2.08) |  | 491 (68.6%) | 1.18 (0.96-1.45) | 1.09 (0.88-1.36) |  | 106 (71.6%) | 0.66 (0.41-1.06) | 0.63 (0.37-1.06) |  | 31 (10.2%) | 1.42 (0.85-2.36) | 1.48 (0.84-2.59) |
|  | Certificate or diploma | 241 (68.3%) | 1.05 (0.75-1.48) | 1.16 (0.79-1.72) |  | 887 (61.0%) | 0.85 (0.72-1.00) | 0.93 (0.78-1.12) |  | 323 (77.1%) | 0.88 (0.59-1.29) | 0.71 (0.46-1.09) |  | 45 (6.0%) | 0.81 (0.51-1.28) | 1.01 (0.61-1.69) |
|  | University degree or higher | 184 (68.7%) | 1.07 (0.75-1.54) | 0.87 (0.56-1.35) |  | 722 (65.0%) | 1.01 (0.84-1.20) | 0.87 (0.71-1.06) |  | 232 (81.7%) | 1.16 (0.75-1.80) | 0.94 (0.57-1.56) |  | 31 (5.8%) | 0.78 (0.47-1.29) | 1.03 (0.58-1.83) |
| **Annual household income (AUD)** | |  |  |  |  |  |  |  |  |  |  |  |  |  |  |  |
|  | Less than $30,000 | 467 (68.6%) | 1 | 1 |  | 1656 (64.3%) | 1 | 1 |  | 480 (74.4%) | 1 | 1 |  | 100 (7.9%) | 1 | 1 |
|  | $30,000-$49,999 | 182 (75.2%) | 1.39 (1.00-1.94) | 1.39 (0.94-2.05) |  | 597 (66.5%) | 1.10 (0.94-1.29) | 1.01 (0.85-1.21) |  | 188 (76.7%) | 1.13 (0.80-1.60) | 1.11 (0.74-1.67) |  | 22 (5.3%) | 0.64 (0.40-1.03) | 0.61 (0.36-1.04) |
|  | $50,000-$69,999 | 87 (64.9%) | 0.85 (0.57-1.25) | 0.74 (0.47-1.18) |  | 351 (67.2%) | 1.14 (0.93-1.39) | 1.00 (0.80-1.26) |  | 108 (83.7%) | 1.77 (1.07-2.91) | 1.70 (0.94-3.07) |  | 13 (5.4%) | 0.67 (0.37-1.21) | 0.69 (0.34-1.39) |
|  | $70,000 or more | 153 (70.5%) | 1.10 (0.78-1.53) | 0.89 (0.56-1.43) |  | 603 (69.0%) | 1.23 (1.05-1.46) | 1.11 (0.89-1.38) |  | 168 (80.0%) | 1.38 (0.94-2.01) | 1.12 (0.66-1.89) |  | 27 (7.4%) | 0.92 (0.59-1.43) | 1.03 (0.55-1.92) |
|  | Won’t disclose | 159 (70.7%) | 1.10 (0.79-1.53) | 0.98 (0.68-1.42) |  | 632 (65.8%) | 1.07 (0.91-1.25) | 0.97 (0.82-1.15) |  | 215 (79.3%) | 1.32 (0.94-1.86) | 1.19 (0.81-1.74) |  | 36 (7.5%) | 0.94 (0.63-1.40) | 1.11 (0.71-1.72) |
| **Working status** | |  |  |  |  |  |  |  |  |  |  |  |  |  |  |  |
|  | Not working | 704 (68.4%) | 1 | 1 |  | 2454 (63.1%) | 1 | 1 |  | 778 (77.1%) | 1 | 1 |  | 137 (7.0%) | 1 | 1 |
|  | Part-time | 188 (69.1%) | 1.03 (0.77-1.38) | 0.83 (0.58-1.18) |  | 744 (65.1%) | 1.10 (0.95-1.26) | 1.14 (0.97-1.34) |  | 223 (78.0%) | 1.05 (0.77-1.44) | 0.72 (0.49-1.06) |  | 35 (7.2%) | 1.04 (0.71-1.53) | 1.41 (0.89-2.23) |
|  | Full-time | 212 (69.7%) | 1.06 (0.81-1.40) | 0.86 (0.58-1.28) |  | 826 (68.9%) | 1.30 (1.13-1.49) | 1.32 (1.10-1.60) |  | 254 (79.1%) | 1.13 (0.83-1.53) | 0.71 (0.46-1.12) |  | 38 (7.1%) | 1.02 (0.70-1.48) | 1.49 (0.86-2.57) |
| **Private health insurance** | |  |  |  |  |  |  |  |  |  |  |  |  |  |  |  |
|  | None of these | 158 (65.0%) | 1 | 1 |  | 630 (66.4%) | 1 | 1 |  | 194 (77.6%) | 1 | 1 |  | 34 (7.7%) | 1 | 1 |
|  | Private health insurance | 575 (70.2%) | 1.27 (0.94-1.72) | 1.18 (0.84-1.65) |  | 2213 (67.0%) | 1.03 (0.88-1.20) | 0.95 (0.81-1.12) |  | 651 (77.7%) | 1.00 (0.72-1.41) | 1.14 (0.78-1.66) |  | 102 (6.8%) | 0.87 (0.58-1.30) | 0.79 (0.51-1.24) |
|  | Concession card | 353 (67.6%) | 1.12 (0.82-1.55) | 1.35 (0.93-1.97) |  | 1192 (59.9%) | 0.76 (0.64-0.89) | 0.90 (0.75-1.08) |  | 409 (77.9%) | 1.02 (0.71-1.46) | 1.20 (0.79-1.83) |  | 73 (7.1%) | 0.91 (0.60-1.40) | 0.78 (0.48-1.28) |
| **SEIFA quintile** | |  |  |  |  |  |  |  |  |  |  |  |  |  |  |  |
|  | Quintile 1 (low SES) | 111 (61.7%) | 1 | 1 |  | 388 (60.0%) | 1 | 1 |  | 114 (74.5%) | 1 | 1 |  | 29 (10.1%) | 1 | 1 |
|  | Quintile 2 | 191 (69.0%) | 1.38 (0.93-2.05) | 1.28 (0.84-1.96) |  | 716 (63.4%) | 1.15 (0.95-1.41) | 1.15 (0.94-1.41) |  | 238 (78.8%) | 1.27 (0.81-2.01) | 1.43 (0.88-2.34) |  | 35 (6.2%) | 0.58 (0.35-0.98) | 0.58 (0.34-1.01) |
|  | Quintile 3 | 475 (68.8%) | 1.37 (0.98-1.93) | 1.48 (1.02-2.15) |  | 1755 (64.0%) | 1.19 (1.00-1.41) | 1.26 (1.05-1.51) |  | 533 (78.5%) | 1.25 (0.83-1.88) | 1.26 (0.82-1.96) |  | 94 (7.6%) | 0.73 (0.47-1.13) | 0.75 (0.47-1.22) |
|  | Quintile 4 | 216 (69.5%) | 1.41 (0.96-2.08) | 1.40 (0.92-2.13) |  | 800 (68.4%) | 1.45 (1.19-1.77) | 1.40 (1.14-1.73) |  | 230 (75.9%) | 1.08 (0.69-1.69) | 1.12 (0.69-1.83) |  | 40 (6.9%) | 0.66 (0.40-1.08) | 0.64 (0.37-1.10) |
|  | Quintile 5 (high SES) | 131 (72.8%) | 1.66 (1.07-2.59) | 1.61 (0.99-2.64) |  | 464 (66.7%) | 1.34 (1.07-1.67) | 1.25 (0.98-1.58) |  | 169 (77.2%) | 1.16 (0.71-1.87) | 1.37 (0.80-2.34) |  | 17 (4.4%) | 0.41 (0.22-0.76) | 0.37 (0.19-0.73) |
| **Remoteness** | |  |  |  |  |  |  |  |  |  |  |  |  |  |  |  |
|  | Major cities | 471 (71.6%) | 1 | 1 |  | 1727 (68.9%) | 1 | 1 |  | 471 (76.0%) | 1 | 1 |  | 72 (6.2%) | 1 | 1 |
|  | Inner regional | 467 (68.5%) | 0.86 (0.68-1.09) | 0.92 (0.71-1.19) |  | 1725 (63.6%) | 0.79 (0.70-0.89) | 0.87 (0.77-0.98) |  | 570 (77.2%) | 1.07 (0.83-1.38) | 1.00 (0.76-1.33) |  | 101 (7.5%) | 1.23 (0.90-1.68) | 1.37 (0.97-1.92) |
|  | Outer regional, remote, very remote | 186 (62.4%) | 0.66 (0.49-0.88) | 0.67 (0.49-0.92) |  | 671 (57.6%) | 0.61 (0.53-0.71) | 0.59 (0.51-0.69) |  | 243 (81.5%) | 1.40 (0.99-1.97) | 1.33 (0.91-1.94) |  | 42 (7.4%) | 1.20 (0.81-1.78) | 1.31 (0.85-2.02) |
| **Number of healthy behaviours** | |  |  |  |  |  |  |  |  |  |  |  |  |  |  |  |
|  | None | 35 (59.3%) | 1 | 1 |  | 119 (61.7%) | 1 | 1 |  | 38 (82.6%) | 1 | 1 |  | 6 (6.7%) | 1 | 1 |
|  | One | 254 (65.5%) | 1.30 (0.74-2.28) | 1.78 (0.96-3.32) |  | 867 (59.1%) | 0.90 (0.66-1.23) | 1.08 (0.78-1.49) |  | 262 (76.2%) | 0.67 (0.30-1.50) | 0.53 (0.22-1.25) |  | 52 (7.7%) | 1.16 (0.48-2.78) | 1.52 (0.59-3.87) |
|  | Two | 418 (68.2%) | 1.47 (0.85-2.54) | 1.77 (0.96-3.25) |  | 1542 (65.8%) | 1.20 (0.89-1.62) | 1.21 (0.88-1.66) |  | 460 (77.3%) | 0.72 (0.33-1.57) | 0.59 (0.25-1.36) |  | 77 (6.9%) | 1.02 (0.43-2.41) | 1.36 (0.54-3.38) |
|  | Three or four | 340 (72.6%) | 1.78 (1.02-3.08) | 2.09 (1.13-3.87) |  | 1230 (66.5%) | 1.26 (0.93-1.70) | 1.21 (0.88-1.66) |  | 404 (79.1%) | 0.75 (0.34-1.64) | 0.59 (0.25-1.37) |  | 80 (6.8%) | 1.00 (0.42-2.37) | 1.38 (0.55-3.45) |
| **BMI** | |  |  |  |  |  |  |  |  |  |  |  |  |  |  |  |
|  | Under-weight | 12 (66.7%) | 0.85 (0.31-2.31) | 0.84 (0.29-2.45) |  | 58 (65.9%) | 0.99 (0.63-1.55) | 1.09 (0.69-1.73) |  | 20 (64.5%) | 0.50 (0.23-1.06) | 0.47 (0.20-1.08) |  | 5 (9.4%) | 1.39 (0.54-3.60) | 1.56 (0.56-4.39) |
|  | Normal weight | 352 (70.1%) | 1 | 1 |  | 1281 (66.1%) | 1 | 1 |  | 433 (78.6%) | 1 | 1 |  | 69 (7.0%) | 1 | 1 |
|  | Over-weight | 370 (70.9%) | 1.04 (0.79-1.36) | 0.96 (0.72-1.28) |  | 1304 (64.4%) | 0.92 (0.81-1.05) | 0.90 (0.79-1.04) |  | 380 (74.5%) | 0.80 (0.60-1.06) | 0.91 (0.67-1.24) |  | 64 (6.6%) | 0.95 (0.67-1.35) | 0.78 (0.53-1.15) |
|  | Obese | 307 (66.9%) | 0.86 (0.66-1.13) | 0.94 (0.68-1.29) |  | 1094 (61.6%) | 0.82 (0.72-0.94) | 0.99 (0.85-1.15) |  | 331 (80.1%) | 1.10 (0.80-1.51) | 1.20 (0.83-1.73) |  | 57 (7.1%) | 1.02 (0.71-1.47) | 0.96 (0.63-1.47) |
| **Self-rated general health** | |  |  |  |  |  |  |  |  |  |  |  |  |  |  |  |
|  | Excellent | 81 (66.4%) | 1 | 1 |  | 399 (64.6%) | 1 | 1 |  | 142 (82.6%) | 1 | 1 |  | 20 (7.0%) | 1 | 1 |
|  | Very good | 297 (72.3%) | 1.32 (0.86-2.03) | 1.54 (0.97-2.46) |  | 1162 (66.7%) | 1.10 (0.91-1.33) | 1.15 (0.94-1.41) |  | 354 (77.6%) | 0.73 (0.47-1.15) | 0.69 (0.43-1.12) |  | 59 (7.1%) | 1.01 (0.60-1.71) | 1.01 (0.57-1.80) |
|  | Good | 388 (69.9%) | 1.18 (0.77-1.78) | 1.41 (0.88-2.26) |  | 1412 (67.8%) | 1.16 (0.96-1.39) | 1.26 (1.03-1.55) |  | 436 (76.8%) | 0.70 (0.45-1.08) | 0.63 (0.39-1.04) |  | 71 (6.7%) | 0.95 (0.57-1.60) | 0.89 (0.49-1.60) |
|  | Fair | 232 (67.4%) | 1.05 (0.68-1.63) | 1.27 (0.75-2.17) |  | 741 (63.2%) | 0.94 (0.77-1.16) | 1.14 (0.91-1.44) |  | 195 (73.6%) | 0.59 (0.36-0.95) | 0.59 (0.34-1.03) |  | 40 (8.2%) | 1.18 (0.68-2.07) | 1.04 (0.53-2.01) |
|  | Poor | 75 (54.7%) | 0.61 (0.37-1.01) | 1.09 (0.56-2.14) |  | 238 (47.4%) | 0.49 (0.39-0.63) | 0.88 (0.64-1.20) |  | 99 (84.6%) | 1.16 (0.61-2.20) | 0.97 (0.39-2.43) |  | 15 (6.1%) | 0.86 (0.43-1.73) | 0.83 (0.33-2.06) |
| **Psychological distress** | |  |  |  |  |  |  |  |  |  |  |  |  |  |  |  |
|  | Low psychological distress | 608 (72.0%) | 1 | 1 |  | 2237 (66.5%) | 1 | 1 |  | 690 (77.1%) | 1 | 1 |  | 114 (7.0%) | 1 | 1 |
|  | Moderate | 209 (68.1%) | 0.83 (0.63-1.10) | 0.92 (0.67-1.26) |  | 738 (64.6%) | 0.92 (0.80-1.06) | 1.00 (0.86-1.16) |  | 223 (81.7%) | 1.32 (0.94-1.87) | 1.35 (0.93-1.97) |  | 35 (6.5%) | 0.92 (0.62-1.37) | 0.91 (0.59-1.40) |
|  | High | 99 (63.9%) | 0.69 (0.48-0.99) | 0.61 (0.40-0.92) |  | 345 (64.4%) | 0.91 (0.75-1.10) | 1.10 (0.90-1.36) |  | 107 (74.3%) | 0.86 (0.57-1.29) | 0.87 (0.54-1.38) |  | 16 (6.3%) | 0.90 (0.53-1.55) | 0.93 (0.51-1.70) |
|  | Very high | 54 (50.0%) | 0.39 (0.26-0.59) | 0.42 (0.24-0.74) |  | 224 (47.7%) | 0.46 (0.38-0.56) | 0.92 (0.71-1.20) |  | 98 (83.1%) | 1.46 (0.88-2.41) | 1.06 (0.50-2.24) |  | 11 (5.2%) | 0.73 (0.39-1.38) | 0.89 (0.37-2.14) |
| **Number of health conditions** | |  |  |  |  |  |  |  |  |  |  |  |  |  |  |  |
|  | Nil | 189 (74.1%) | 1 | 1 |  | 726 (66.8%) | 1 | 1 |  | 211 (81.2%) | 1 | 1 |  | 29 (5.9%) | 1 | 1 |
|  | One | 262 (70.8%) | 0.85 (0.59-1.21) | 0.80 (0.54-1.17) |  | 928 (66.2%) | 0.97 (0.82-1.15) | 0.96 (0.81-1.15) |  | 334 (80.3%) | 0.95 (0.64-1.40) | 1.02 (0.66-1.56) |  | 53 (7.7%) | 1.32 (0.83-2.11) | 1.23 (0.74-2.04) |
|  | Two | 244 (70.3%) | 0.83 (0.58-1.19) | 0.82 (0.55-1.23) |  | 901 (67.5%) | 1.03 (0.87-1.22) | 1.01 (0.84-1.21) |  | 279 (74.6%) | 0.68 (0.46-1.01) | 0.76 (0.49-1.17) |  | 55 (8.2%) | 1.42 (0.89-2.26) | 1.26 (0.74-2.13) |
|  | 3 or 4 | 290 (65.5%) | 0.66 (0.47-0.93) | 0.69 (0.46-1.04) |  | 1116 (64.0%) | 0.88 (0.75-1.03) | 0.89 (0.74-1.07) |  | 298 (74.3%) | 0.67 (0.46-0.99) | 0.76 (0.48-1.20) |  | 49 (6.1%) | 1.03 (0.64-1.66) | 0.81 (0.46-1.41) |
|  | 5+ | 139 (62.3%) | 0.58 (0.39-0.85) | 0.80 (0.48-1.31) |  | 452 (55.5%) | 0.62 (0.51-0.75) | 0.86 (0.68-1.08) |  | 162 (79.0%) | 0.87 (0.55-1.38) | 0.76 (0.43-1.36) |  | 29 (7.1%) | 1.23 (0.72-2.09) | 1.22 (0.63-2.36) |
| **Number of medications taken** | |  |  |  |  |  |  |  |  |  |  |  |  |  |  |  |
|  | Nil | 376 (69.5%) | 1 | 1 |  | 1441 (66.3%) | 1 | 1 |  | 479 (79.8%) | 1 | 1 |  | 65 (6.0%) | 1 | 1 |
|  | One | 249 (73.9%) | 1.24 (0.92-1.68) | 1.26 (0.90-1.75) |  | 969 (65.8%) | 0.98 (0.85-1.13) | 0.99 (0.85-1.14) |  | 292 (76.4%) | 0.82 (0.60-1.12) | 0.97 (0.69-1.36) |  | 56 (8.2%) | 1.40 (0.97-2.03) | 1.36 (0.91-2.03) |
|  | Two | 175 (62.9%) | 0.75 (0.55-1.01) | 0.77 (0.54-1.09) |  | 696 (67.1%) | 1.04 (0.89-1.22) | 1.00 (0.84-1.19) |  | 198 (73.9%) | 0.71 (0.51-1.00) | 0.96 (0.65-1.41) |  | 39 (7.8%) | 1.32 (0.87-1.99) | 1.18 (0.74-1.88) |
|  | 3 or 4 | 226 (68.3%) | 0.94 (0.70-1.27) | 0.98 (0.69-1.40) |  | 747 (64.7%) | 0.93 (0.80-1.09) | 0.92 (0.77-1.09) |  | 216 (76.9%) | 0.84 (0.60-1.18) | 1.09 (0.73-1.63) |  | 35 (6.4%) | 1.07 (0.70-1.64) | 0.90 (0.55-1.47) |
|  | 5+ | 98 (64.9%) | 0.81 (0.55-1.19) | 1.35 (0.82-2.22) |  | 270 (49.5%) | 0.50 (0.41-0.60) | 0.69 (0.54-0.87) |  | 99 (79.2%) | 0.96 (0.60-1.55) | 0.84 (0.46-1.55) |  | 20 (7.7%) | 1.30 (0.77-2.18) | 1.49 (0.78-2.86) |
| **Time of call** | |  |  |  |  |  |  |  |  |  |  |  |  |  |  |  |
|  | In- hours | 239 (63.2%) | 1 | 1 |  | 1190 (63.6%) | 1 | 1 |  | 395 (79.5%) | 1 | 1 |  | 50 (5.0%) | 1 | 1 |
|  | After-hours | 885 (70.2%) | 1.37 (1.08-1.75) | 1.46 (1.12-1.89) |  | 2933 (65.0%) | 1.06 (0.95-1.19) | 1.07 (0.95-1.20) |  | 889 (76.7%) | 0.85 (0.66-1.10) | 0.76 (0.57-1.00) |  | 165 (8.0%) | 1.65 (1.19-2.29) | 1.56 (1.10-2.20) |
| **Caller-patient relationship** | |  |  |  |  |  |  |  |  |  |  |  |  |  |  |  |
|  | Self | 819 (67.5%) | 1 | 1 |  | 3320 (63.2%) | 1 | 1 |  | 1128 (78.1%) | 1 | 1 |  | 170 (6.3%) | 1 | 1 |
|  | Partner | 188 (72.9%) | 1.30 (0.96-1.75) | 1.25 (0.88-1.77) |  | 453 (71.6%) | 1.47 (1.22-1.76) | 1.29 (1.06-1.57) |  | 87 (72.5%) | 0.74 (0.49-1.12) | 0.97 (0.60-1.57) |  | 19 (9.6%) | 1.59 (0.96-2.61) | 1.14 (0.65-2.00) |
|  | Others | 106 (69.7%) | 1.11 (0.77-1.60) | 1.27 (0.83-1.95) |  | 316 (71.7%) | 1.47 (1.19-1.83) | 1.33 (1.06-1.68) |  | 57 (73.1%) | 0.76 (0.45-1.27) | 0.97 (0.55-1.70) |  | 24 (17.4%) | 3.15 (1.97-5.02) | 2.36 (1.38-4.03) |
| **Original intention** | |  |  |  |  |  |  |  |  |  |  |  |  |  |  |  |
|  | Self-care at home | 134 (55.1%) | 1 | 1 |  | 499 (56.1%) | 1 | 1 |  | 286 (79.9%) | 1 | 1 |  | 30 (5.1%) | 1 | 1 |
|  | Call ambulance or attend ED | 397 (76.5%) | 2.65 (1.91-3.66) | 2.59 (1.82-3.69) |  | 886 (70.0%) | 1.82 (1.52-2.18) | 1.69 (1.40-2.03) |  | 115 (72.8%) | 0.67 (0.44-1.04) | 0.75 (0.47-1.20) |  | 52 (15.9%) | 3.47 (2.17-5.57) | 2.64 (1.59-4.38) |
|  | Contact doctor or health provider | 155 (64.3%) | 1.47 (1.02-2.11) | 1.36 (0.92-2.02) |  | 1156 (67.8%) | 1.64 (1.39-1.94) | 1.56 (1.31-1.86) |  | 243 (74.1%) | 0.72 (0.50-1.03) | 0.78 (0.53-1.14) |  | 48 (6.7%) | 1.32 (0.83-2.12) | 1.18 (0.72-1.94) |
|  | Did not know what to do | 373 (69.2%) | 1.83 (1.34-2.50) | 1.88 (1.34-2.63) |  | 1328 (62.5%) | 1.30 (1.11-1.53) | 1.27 (1.08-1.50) |  | 524 (79.3%) | 0.96 (0.70-1.33) | 1.01 (0.72-1.42) |  | 67 (5.8%) | 1.13 (0.73-1.76) | 1.04 (0.65-1.64) |
| **Clinical guidelines** | |  |  |  |  |  |  |  |  |  |  |  |  |  |  |  |
|  | Skin, wound | 50 (63.3%) | 1 | 1 |  | 329 (64.3%) | 1 | 1 |  | 41 (71.9%) | 1 | 1 |  | 5 (4.6%) | 1 | 1 |
|  | Limbs and extremities | 81 (68.6%) | 1.27 (0.70-2.31) | 1.13 (0.60-2.15) |  | 483 (65.4%) | 1.05 (0.83-1.33) | 1.21 (0.95-1.54) |  | 103 (81.7%) | 1.75 (0.84-3.64) | 1.69 (0.79-3.62) |  | 5 (2.3%) | 0.80 (0.19-3.42) | 0.75 (0.17-3.26) |
|  | Bite, burns, chemical exposure | 65 (60.7%) | 0.90 (0.49-1.64) | 0.83 (0.43-1.59) |  | 181 (63.5%) | 0.97 (0.72-1.31) | 1.02 (0.75-1.39) |  | 201 (82.7%) | 1.87 (0.96-3.64) | 1.95 (0.97-3.90) |  | 7 (2.7%) | 0.97 (0.25-3.82) | 0.84 (0.21-3.38) |
|  | Respiratory | 65 (63.1%) | 0.99 (0.54-1.82) | 1.00 (0.52-1.92) |  | 235 (64.6%) | 1.01 (0.77-1.34) | 1.03 (0.77-1.37) |  | 37 (82.2%) | 1.80 (0.69-4.70) | 1.85 (0.68-5.07) |  | 5 (5.7%) | 1.24 (0.24-6.31) | 1.06 (0.20-5.60) |
|  | Head, neck, face non-injury | 46 (68.7%) | 1.27 (0.64-2.53) | 1.17 (0.56-2.44) |  | 179 (70.2%) | 1.31 (0.95-1.81) | 1.42 (1.01-1.98) |  | 22 (81.5%) | 1.72 (0.55-5.31) | 1.82 (0.56-5.88) |  | 6 (4.4%) | 1.57 (0.38-6.44) | 1.63 (0.39-6.83) |
|  | Neurological, headache, seizure | 37 (63.8%) | 1.02 (0.51-2.07) | 0.90 (0.42-1.93) |  | 442 (64.5%) | 1.01 (0.80-1.29) | 1.03 (0.81-1.32) |  | 24 (61.5%) | 0.62 (0.26-1.48) | 0.66 (0.27-1.65) |  | 10 (6.9%) | 2.56 (0.69-9.55) | 2.23 (0.58-8.54) |
|  | Abdominal pain or injury | 150 (70.4%) | 1.38 (0.80-2.38) | 1.18 (0.66-2.11) |  | 406 (69.6%) | 1.28 (0.99-1.64) | 1.39 (1.07-1.80) |  | 16 (80.0%) | 1.56 (0.45-5.39) | 1.47 (0.41-5.28) |  | 7 (5.3%) | 1.91 (0.48-7.56) | 2.02 (0.50-8.21) |
|  | Cold, flu, fever | 17 (70.8%) | 1.41 (0.52-3.80) | 1.51 (0.52-4.32) |  | 173 (61.6%) | 0.89 (0.66-1.20) | 0.92 (0.67-1.25) |  | 49 (74.2%) | 1.12 (0.51-2.50) | 1.11 (0.48-2.56) |  | 6 (7.1%) | 2.64 (0.64-10.89) | 2.10 (0.49-9.01) |
|  | Postoperative | 33 (76.7%) | 1.91 (0.82-4.44) | 1.83 (0.75-4.45) |  | 207 (66.1%) | 1.09 (0.81-1.46) | 1.12 (0.83-1.52) |  | 41 (78.8%) | 1.45 (0.60-3.51) | 1.67 (0.67-4.18) |  | 7 (9.0%) | 3.38 (0.85-13.53) | 2.51 (0.61-10.38) |
|  | Nausea, vomiting | 41 (78.8%) | 2.16 (0.96-4.84) | 1.76 (0.75-4.12) |  | 106 (58.2%) | 0.78 (0.55-1.10) | 0.72 (0.51-1.03) |  | 48 (76.2%) | 1.25 (0.55-2.83) | 1.51 (0.64-3.59) |  | 10 (12.5%) | 4.88 (1.45-16.47) | 3.91 (1.00-15.27) |
|  | Cardiac | 261 (72.5%) | 1.53 (0.92-2.55) | 1.45 (0.84-2.51) |  | 264 (62.7%) | 0.94 (0.72-1.22) | 0.94 (0.71-1.24) |  | 22 (66.7%) | 0.78 (0.31-1.97) | 0.81 (0.31-2.13) |  | 27 (12.4%) | 5.28 (1.49-18.67) | 3.94 (1.14-13.63) |
|  | Bleeding | 61 (74.4%) | 1.68 (0.86-3.31) | 1.88 (0.91-3.86) |  | 220 (72.1%) | 1.44 (1.06-1.96) | 1.40 (1.02-1.91) |  | 26 (72.2%) | 1.01 (0.40-2.57) | 1.12 (0.42-2.98) |  | 16 (13.3%) | 4.90 (1.30-18.46) | 5.15 (1.41-18.75) |
|  | Gastrointestinal | 40 (59.7%) | 0.86 (0.44-1.68) | 0.86 (0.42-1.77) |  | 181 (57.3%) | 0.75 (0.56-0.99) | 0.73 (0.55-0.99) |  | 64 (81.0%) | 1.67 (0.74-3.73) | 2.02 (0.88-4.66) |  | 40 (14.2%) | 5.67 (1.72-18.76) | 4.81 (1.42-16.28) |
|  | Head, neck, face injury | 34 (61.8%) | 0.94 (0.46-1.91) | 0.87 (0.41-1.87) |  | 91 (64.5%) | 1.01 (0.69-1.49) | 1.03 (0.69-1.54) |  | 36 (75.0%) | 1.17 (0.49-2.80) | 1.32 (0.53-3.26) |  | 10 (13.3%) | 5.28 (1.40-19.91) | 5.17 (1.33-20.04) |
|  | Seen by a provider earlier |  |  |  |  | 199 (67.9%) | 1.18 (0.87-1.60) | 1.22 (0.89-1.67) |  | 115 (63.9%) | 0.69 (0.36-1.33) | 0.67 (0.34-1.32) |  | 25 (9.3%) | 3.53 (1.04-11.96) | 3.60 (1.04-12.51) |
|  | Other symptoms | 143 (68.1%) | 1.24 (0.72-2.13) | 1.18 (0.66-2.12) |  | 427 (60.1%) | 0.84 (0.66-1.06) | 0.92 (0.72-1.17) |  | 439 (81.0%) | 1.66 (0.90-3.08) | 1.68 (0.88-3.19) |  | 29 (3.7%) | 1.51 (0.46-5.02) | 1.44 (0.42-4.88) |

*: Among patients given dispositions including self-care, see doctor in 72 hours or 2 weeks, and see a dentist or a health provider in 72 hours, 2 weeks or when available

**: Adjusted for patient age at call, sex, country of birth, marital status, number of people that the patient can depend on, education, household income, working status, private health insurance, SEIFA, number of healthy behaviours, BMI, self-rated general health,
 psychological distress, number of medications taken, time of call, caller-patient relationship, original intention, and triage protocols. Models examining remoteness did not include SEIFA and models examining morbidity did not include the use of medications variable.
 Missing information was treated as a separate category for any variables with missing data (data not shown).
